# Supplementary material for: Field experiments to assess passage of juvenile salmonids across beaver dams during low flow conditions in a tributary to the Klamath River, California, USA
Source: PLoS One. 2022 May 24;17(5):e0268088. doi: 10.1371/journal.pone.0268088 (PMC9129030; doi:10.1371/journal.pone.0268088)
Supplement: Attachment — Submitted filename: PONE-Fish Passage response to reviewers.docx [file pone.0268088.s002.docx]

PONE-D-19-32503
A field experiment to assess passage of juvenile salmonids across beaver dams during low flow conditions in a tributary to the Klamath River, California, USA.
PLOS ONE

Dear Dr. Pollock,

Thank you for submitting your manuscript to PLOS ONE. After careful consideration, we feel that it has merit but does not fully meet PLOS ONE’s publication criteria as it currently stands. Therefore, we invite you to submit a revised version of the manuscript that addresses the points raised during the review process. Please see the extensive comments from two reviewers.

We would appreciate receiving your revised manuscript by Mar 02 2020 11:59PM. When you are ready to submit your revision, log on to <https://www.editorialmanager.com/pone/> and select the 'Submissions Needing Revision' folder to locate your manuscript file.

If you would like to make changes to your financial disclosure, please include your updated statement in your cover letter.

To enhance the reproducibility of your results, we recommend that if applicable you deposit your laboratory protocols in [protocols.io](http://protocols.io), where a protocol can be assigned its own identifier (DOI) such that it can be cited independently in the future. For instructions see: <http://journals.plos.org/plosone/s/submission-guidelines#loc-laboratory-protocols>

Please include the following items when submitting your revised manuscript:

- A rebuttal letter that responds to each point raised by the academic editor and reviewer(s). This letter should be uploaded as separate file and labeled 'Response to Reviewers'.
- A marked-up copy of your manuscript that highlights changes made to the original version. This file should be uploaded as separate file and labeled 'Revised Manuscript with Track Changes'.
- An unmarked version of your revised paper without tracked changes. This file should be uploaded as separate file and labeled 'Manuscript'.

Please note while forming your response, if your article is accepted, you may have the opportunity to make the peer review history publicly available. The record will include editor decision letters (with reviews) and your responses to reviewer comments. If eligible, we will contact you to opt in or out.

We look forward to receiving your revised manuscript.

Kind regards,

Madison Powell, PhD
Academic Editor
PLOS ONE


Additional Editor Comments (if provided):

The manuscript provides a convincing conclusion albeit with data analyses and discussions that can be much improved. Even though the conclusions are relatively clear, they need to be treated more rigorously to rise to the level of publication. Despite the sub-par execution on the manuscript, there are sufficient informative data to publish provided extensive changes are made. Both reviewers have provided the authors with the advantage of very insightful and thorough reviews of their original submission. The authors are strongly encouraged to follow the comments, extensively revise their manuscript and resubmit these data.

Journal Requirements:

When submitting your revision, we need you to address these additional requirements.

1. Please ensure that your manuscript meets PLOS ONE's style requirements, including those for file naming. The PLOS ONE style templates can be found at <http://www.journals.plos.org/plosone/s/file?id=wjVg/PLOSOne_formatting_sample_main_body.pdf> and <http://www.journals.plos.org/plosone/s/file?id=ba62/PLOSOne_formatting_sample_title_authors_affiliations.pdf>

2. In your Methods section, please provide additional location information of the study area, including geographic coordinates for the data set if available.

All data are available at NOAA’s Northwest Fisheries Science Center in Seattle, Washington, USA (<https://www.nwfsc.noaa.gov/>, UTM coordinates = 10T 552099E, 5277064N).

We included the UTM coordinates of Sugar Creek study area as well.

3. We note that Figures 1 and 2 in your submission contain map and satellite images which may be copyrighted. All PLOS content is published under the Creative Commons Attribution License (CC BY 4.0), which means that the manuscript, images, and Supporting Information files will be freely available online, and any third party is permitted to access, download, copy, distribute, and use these materials in any way, even commercially, with proper attribution. For these reasons, we cannot publish previously copyrighted maps or satellite images created using proprietary data, such as Google software (Google Maps, Street View, and Earth). For more information, see our copyright guidelines: <http://journals.plos.org/plosone/s/licenses-and-copyright>.

 Authors Response: Figure 1 does not contain any copyrighted information. It is public domain data. The copyrighted Google Earth images from Figure 2 have been removed and replaced with a schematic.

4. Thank you for stating the following in the Acknowledgments Section of your manuscript:

" This project was funded internally at the NOAA’s Northwest Fisheries Science Center, NOAA’s California
Coastal Office, Klamath Branch, and the NOAA Restoration Center."

We note that you have provided funding information that is not currently declared in your Funding Statement. However, funding information should not appear in the Acknowledgments section or other areas of your manuscript. We will only publish funding information present in the Funding Statement section of the online submission form.

Please remove any funding-related text from the manuscript and let us know how you would like to update your Funding Statement. Currently, your Funding Statement reads as follows:

Enter: The author(s) received no specific funding for this work.

Authors response: The funding statement has been removed from Acknowledgements. Please update the Funding Statement to read: “This project was funded internally at the NOAA’s Northwest Fisheries Science Center, NOAA’s California Coastal Office, Klamath Branch, and the NOAA Restoration Center.”

5. We note that you have indicated that data from this study are available upon request. PLOS only allows data to be available upon request if there are legal or ethical restrictions on sharing data publicly. For more information on unacceptable data access restrictions, please see <http://journals.plos.org/plosone/s/data-availability#loc-unacceptable-data-access-restrictions>.

In your revised cover letter, please address the following prompts:

a) If there are ethical or legal restrictions on sharing a de-identified data set, please explain them in detail (e.g., data contain potentially sensitive information, data are owned by a third-party organization, etc.) and who has imposed them (e.g., an ethics committee). Please also provide contact information for a data access committee, ethics committee, or other institutional body to which data requests may be sent.

b) If there are no restrictions, please upload the minimal anonymized data set necessary to replicate your study findings as either Supporting Information files or to a stable, public repository and provide us with the relevant URLs, DOIs, or accession numbers. For a list of acceptable repositories, please see <http://journals.plos.org/plosone/s/data-availability#loc-recommended-repositories>.

We will update your Data Availability statement on your behalf to reflect the information you provide.

The [PLOS Data policy](http://www.plosone.org/static/policies.action#sharing) requires authors to make all data underlying the findings described in their manuscript fully available without restriction, with rare exception (please refer to the Data Availability Statement in the manuscript PDF file). The data should be provided as part of the manuscript or its supporting information, or deposited to a public repository. For example, in addition to summary statistics, the data points behind means, medians and variance measures should be available. If there are restrictions on publicly sharing data—e.g. participant privacy or use of data from a third party—those must be specified.

Authors response: The data are publicly available, there are no restrictions. The data have been uploaded as Supporting Information.

6. Your ethics statement must appear in the Methods section of your manuscript. If your ethics statement is written in any section besides the Methods, please move it to the Methods section and delete it from any other section. Please also ensure that your ethics statement is included in your manuscript, as the ethics section of your online submission will not be published alongside your manuscript.

**Comments to the Author**

Review Comments to the Author

Please use the space provided to explain your answers to the questions above. You may also include additional comments for the author, including concerns about dual publication, research ethics, or publication ethics. (Please upload your review as an attachment if it exceeds 20,000 characters)

Reviewer #1: Review of PONE-D-19-32503
A field experiment to assess passage of juvenile salmonids across beaver dams during low flow conditions in a tributary to the Klamath River, California, USA.

Overall, I found this an interesting study. It has both strengths and weakness, which I will attempt to describe, each in turn.

Strengths: the primary area of interest in relation to this manuscript is, as the authors recognise, this is one of the very few studies that have attempted to quantify fish movements past barriers, whether they be anthropogenic or natural. The introduction of the manuscript is very interesting, and does a great job of putting natural barriers, constructed by beavers, but also log jams, within the context of current day perspectives. I understand what the authors are getting at regarding the advantage of using beavers as a means of providing cost effective and sustainable mechanisms of river restoration (i.e. by restoring processes), but I am unsure as to whether this concept has as yet caught on by the majority of people, as opposed to the few enlightened ones. Certainly, the authors suggest this to be the case in Europe. It is not; reintroduction is taking place throughout Europe primarily to enhance biodiversity (by the addition of another species), with benefits for river restoration being an afterthought, if at all (again with the exception of a few enlightened ones). For example, very few in Europe are using BDAs to help improve river restoration strategies. I also think some caution needs to be given to the comparison between there being fewer dams in North America now than prior to European colonisation. This is true, and I understand the point the authors are making, and they do say “albeit of a different size and materials”, but in reality anthropogenic dams have very different impacts on modified rivers (that are less able to respond to those that historically were in the reference condition). The authors understand this, and discuss it later, but consideration of how such statements might be used by the “pro-dam” lobby is important; they might use it to justify their positions against the removal of some of the anthropogenic North American dams that have considerable environmental impact.

R1 Comment: The Discussion also raises several very interesting and important points. I particularly enjoyed the final section that discussed, philosophically, the relationship (trade-off perhaps) of habitat connectivity and habitat quality, and recognition that there has been too much of a focus on connectivity, while forgetting that biogenic dams are a natural part of our river systems and important driver of adaptation in salmonids. However, I would be very cautious of statements such as: “We speculate that in the case of coho salmon, decades of emphasizing habitat connectivity over habitat quality by removing perceived obstructions to fish passage is a significant contributing factor to their widespread decline”. Clearly, this is speculation, there is no evidence provided to support such a statement, and it is contentious – expect others to challenge this, with evidence based arguments to the contrary. I wonder if such a strong statement is needed, as the text that follows discusses the point eloquently. The final sections call for a more holistic approach that considers both habitat quality and connectivity, and I fully agree.

Author Response: We agree and have softened the language.

R1 Comment: Weaknesses: Overall, the study provides very clear results (i.e. displaced fish can relocate to the former microhabitats by negotiating BDAs, either by jumping over or moving around) from a rather low resolution, unsophisticated PIT telemetry study that utilised a small number of fish. For example, the presentation of the results is rather descriptive, and fails to use techniques commonly used in more traditional “fish passage” studies (e.g. time to event analysis, passage efficiency, approach rate [which can be used as a measure of motivation], time to pass etc.). As mentioned in the “strengths section”, I really enjoyed the study from the perspective of the points discussed in the introduction and discussion, but was a little disappointed by the quality of the PIT telemetry study itself. That said, the result are so clear it hardly matters – the point is made. These two species of salmonids can definitely pass beaver dam analogues.

Author Response: More common fish passage analysis techniques such as time to event, passage efficiency and approach rate are traditionally performed on adult fish that can be observed or tracked with radio transmitters and that must pass through a confined area such as a concrete fishway. We did not have the proper instrumentation to answer such questions. The study wasn’t designed to answer such questions. We had a “leaky” system in that there were numerous flow paths that a fish could follow that we would not detect with our antenna network. Our network was simply set up with antennas placed in places we were likely to detect fish and be able to assess whether they had crossed the dam, and if so, by what route.

The issue was also complicated a bit by the fact that we PIT-tagged the fish just prior to placing them in the release pool, an event that involves capture, anesthetizing, and surgically implanting a 12 mm long tag into the peritoneal cavity of a 65-80 mm long fish, so even if we did have the proper instrumentation to precisely track movement, there may have been a delay in movement resulting from recovery needed after tagging.

The comment as to the quality of the PIT study was unclear. As far as we know, this is the most comprehensively instrumented PIT study of juvenile salmonid passage over an instream obstacle of which we are aware. We used 10 separate antennas across to monitor specific movement patterns in as great a detail as was reasonably possible given the limits and expense of PIT technology. We feel that we have provided substantial insight into the movement and behavior of juvenile salmonids. Many of the questions the reviewer would like answered are interesting, and would require a different technology, such as radiotransmitters attached to fish so that their specific movements could be monitored. We agree that such an experiment would provide interesting data, but we do not have the budget for such a study.

R1 Comment: However, I do have concerns in relation to the measure and subsequent use of the values for efficiency of the telemetry array – these reflect probability of subsequent detection (e.g. 91% for coho) and is not a measure of efficiency. The authors explain that the PIT antennae only provided partial coverage. I would like to know what the probability of a fish passing the entire array being detected is. Some fish will miss the array completely, while PIT tags, while good, are never 100% detected, so even those that pass the antennae might not be detected (e.g. tag failure which is rare these days, but antenna may be malfunctioning, or tag too far away or wrong orientation to be read). The authors describe the ability of the fish to pass up and downstream over the dam and back over again multiple times (potentially). Therefore, a value of 91% might relate to cumulative detection, i.e. a fish could potentially have passed the array 20 times, but recorded only once (unlikely I know, but I’m trying to emphasise the point). In other studies, researchers do a range of things to try and estimate an efficiency of their systems, e.g. towing sticks with tags on through arrays multiple time so that they have a best guess of the efficiency. At the end of the results the authors go on to provide estimates of survival based on these array efficiencies for outmigrating fish. I think this is problematic – the values used were for fish that were able to pass multiple times, potentially under different flow conditions (flow can effect efficiency) as suggested in the discussion, rather than on a uni-direction persistent movement downstream.

Authors Response: We have changed the language to reflect the fact that we did not measure efficiency, but rather probability of detection for the passage study. It would be optimal to have efficiency estimates, but as noted above, the “leaky” nature of the fish passage options makes such an estimate not possible. There is no means by which we can determine how many fish chose a passage route that was not instrumented with an antenna, though it seems clear that steelhead chose alternate routes at a higher rate than coho, based on the large difference in the probability of detection upstream of the dams between the two species.

I found the title slightly misleading. It refers to beaver dams, but having read the manuscript it is clear that these are human made replicates of beaver dams, i.e. beaver dam analogues. It is not clear whether they were then modified by beavers themselves, or whether these actually are anthropogenic structures. If the latter, this needs to be clear in the title.

Authors response: We added language to indicate the role that beaver have played in modifying the structures and that the BDA was built on the site of an abandoned beaver dam, and that it is a hybrid structure built and modified by both humans and beaver.

Also, the study is a series of experiments, not “A field experiment”.

Authors response: We have changed “experiment” to “experiments”

Finally, it is not clear (unless I missed this) how this is a low flow condition, how low was it compared to the mean for the period of year studied?

Authors response: We have added the mean annual discharge of the site into the figure xx caption (the stream hydrograph figure) for comparison to the low flow conditions during which the experiments were performed.

General Comments

The study describes several ‘experiments’ where PIT-tagged fish were monitored upstream and downstream from some artificial beaver dams. The concept was reasonable and the research question – can fish pass these structures? – is relevant for managers and researchers. My overall sense was that the methods were reasonable and some useful datasets and results were generated, although there were no control conditions for any of the experiments. I was also convinced on the main objective – the study fish readily passed the artificial beaver dams. That said, the quality of the data summaries and supporting narrative were not particularly strong.

My primary concerns were: (1) there are a number of ancillary and anecdotal data elements presented that distract from the experimental results; (2) one of the experiments (#3) appears to have used fish from a potentially mixed-origin downstream population that might not have had any reason to attempt upstream movement at the dams in the study tributary; (3) the statistics are very simplistic, with essentially all limited to percentages that passed the dams and that used various routes; there are a number of statistical approaches (i.e., time-to-event passage analyses, passage probability models using fish and environmental covariates like time of day, etc.) that could potentially generate much more informative and quantitative results; and (4) the scientific writing quality was below average. Please see specific comments for examples.

Authors response: these concerns are addressed under specific comments, below.

Specific Comments

1. Title. Not really one experiment

We have changed “experiment” to “experiments”

2. Line 24. Consider using analogue language here, since that was just established

We have included the word analogue

3. Line 26. Clumsy sentence with repeated ambiguous ‘of them’. Also, summer parr is a very specific life history stage identification, but reverts to ‘juvenile’ thereafter. Are these fish still considered ‘summer parr’ late in the fall?

We have removed the term ‘summer parr’ to avoid confusion. We have tightened the sentence structure to clarify the ambiguous use of “of them”.

4. Line 28-29. ‘were’ = ‘had been’? the 1% and 15% numbers are difficult to interpret since it seems like almost all fish would be detected near the release site (and this seems to be the case in the summary tables). Unless this means detected downstream from the block nets, which is certainly not clear as written.

We clarified the language to indicate that these were the fish that were still detected in the release pool at the end of the 21-day experiment.

5. Line 31. Serious concerns about this third experiment which may (?) be fish from another population and might not be expected to move upstream.

This is discussed in the Discussion. We agree that they may be from another population and that they might not be expected to move upstream and have added clarifying language so that the readers better understand that the third experiment was using fish that are likely from another population and that they were not displaced.

6. Line 35. A stronger set of take home messages is needed, since this was not really a study of restoration efforts. Fish could pass upstream and there was no downstream assessment. Could emphasize the low-flow experimental conditions, when presumably passage might be most difficult.

We changed the language to emphasize low flow conditions and that multiple types of routes may be advisable when considering fish passage.

7. Line 88. All examples are salmonids, which communicates an unintended bias in the context of the 48 species mentioned in topic sentence.

We refer to commercially important species that use beaver ponds, all of which happened to be in the family Salmonidae.

8. Line 114. What is a ‘naturalistic situation’?

We removed the term to avoid confusion

9. Line 118. Upstream passage only here – or bidirectional? Might want to provide some justification for including steelhead. Are they also an ESA-listed species? Native species in the study system?

We added clarifying language to indicate upstream movement only and also that steelhead trout a are a native species in the study system.

10. Line 119. Jumping vs swim around objective not mentioned in Abstract.

Abstract sentence changed to ‘we tested the ability of juvenile salmonids to jump over and swim around constructed beaver dams’

11. Line 120. The fish benefit objective was not mentioned in Abstract and seems like mostly an afterthought.

We changed the last sentence of the abstract to read: ‘Finally, we compared estimates of the number of juvenile salmonids using the pond habitat upstream of the dam relative to the number that the dam may have prevented from moving upstream. We found a several orders of magnitude difference in favor of the number of juveniles using the pond habitat upstream of the dam.’

12. Line 126. So was design evaluation a current study objective? Abstract suggest it sort of was, but the threads are pretty thin in Methods and Results.

Design evaluation was not a study objective per se, but we thought the results might be useful to future restoration designs. We changed the sentence as follows to clarify this point: “While this study was not a design evaluation of fish passage structures per se, the results of this study may be useful towards guiding future design considerations for fish passage at stream restoration structures.

13. Line 136. ‘in greater’ ?

This was deleted.

14. Line 141. Wetland rather than swamp? The latter has a fairly specific definition.

We replaced ‘swamp’ with ‘wetland’

15. Line 156. Does water year ‘quality’ differ from snowpack fluctuations in some way?

We deleted the phrase ‘quality of water year’

16. Line 162. Not quite clear how this paragraph is important?

We have deleted this paragraph.

17. Line 180. ‘wiggle through some of the pores’ = ‘move through gaps’. The wiggle language is non-scientific and should be replaced throughout.

We changed ‘wiggled’ to ‘moved’. There was only one use of that term.

18. Line 184. The narrative seems to be slipping into present tense and that is particularly noticeable in this paragraph.

The narrative has been changed to past tense.

19. Line 191. Suggest cutting the word family, unless this was verified or is in some other way important.

The term ‘family’ has been deleted.

20. Line 197. The below-barrier tagging is not really captured in the Abstract.

We included this sentence: A similar but shorter 4-day pilot experiment with only steelhead trout produced similar results. In contrast, in a non-displacement experiment, juveniles of both species that were captured, tagged and released in a pool 50 m below the dams showed little inclination to move upstream.

21. Line 198. Why no interest in downstream movement? Because not perceived as a problem?

We included language to indicate that obstruction of downstream movement was not perceived as a potential problem.

22. Line 200. How were fish collected? Tagging methods?

We included additional language in the Methods section describing how the fish were collected and tagged.

23. Line 206. So which fish were used in experiment 1 – the 758 or the 32+16? The 758 don’t really seem to be in the Results.

We deleted reference to the 758 fishes tagged because they were tangential to the study and were not discussed in the results.

24. Line 209. I suggest separating fish collection and tagging from the monitoring.

We inserted a paragraph break between the description of the fish collection/tagging and the fish monitoring.

25. Figure 2. Image quality is low in the pdf.

The original is high quality. There was some sort of translation error that we have corrected.

26. Lines 243,305. I suggest removing all references to Chinook salmon as they were not described in any other manuscript sections.

We left the reference to Chinook salmon in because it is documentation that they are using the Scott River system during the summer, which has not been previously reported subsequent to the reported extirpation of the spring Chinook salmon run in the late 1970s. We included a brief mention of this in the discussion section.

27. Line 244. What is meant by easing fish passage and why was that needed?

We clarified the language to explain that the additional structures were added to reduce the 60 cm jump height of the original structure into a series of three steps, and that passage of the original structure was focused on the side channel that went around the structure.

28. Line 246. The BDA naming conventions are unnecessarily complex. Why not just 1,2,3 or the intuitive lower, middle, upper?

These numbers have been changed to 1,2 and 3.

29. Line 255. Antenna naming also oddly complex and non-intuitive. 00,09,06,08,05,90,100?

These numbers have been changed to A-1, A-2 etc. excepting that the 90 and 100 terminology for the permanent upstream antennas are retained and a note is included explaining that these number reflect the distance upstream in meters from the mouth of Sugar Creek.

30. Line 262. Relevance of the cottonwood?

This helps illustrate that beaver are actively using the habitat.

31. Line 297. I think these two antennas have been described three times.

We deleted this reference to the two permanent antennas.

32. Line 346. Nesting experiment 3-5 within Experiment 3 is confusing.

We changed the terminology from 3,4 and 5 to 3.1, 3.2 and 3.3

33. Line 308. Ambiguous ‘them’. Might also state upstream movement.

Clarified to remove the ambiguity and to indicate upstream movement

34. Line 310. What was the reason for these population estimates? Not mentioned in Abstract or in the objectives statements in the Introduction.

We have included clarification in the Abstract and in the objectives statement of the Introduction

35. Line 323. Please report percentages and sample sizes rather than the ‘n of the n’ format throughout the results.

We have changed the format to reflect percentages and sample sizes

36. Line 329, 333. The anecdote about the three fish and the one fish are not very useful.

We thought that these data add to the central finding that juvenile salmonids are able to transit these obstructions with relative ease. We prefer that these data remain in the manuscript.

37. Line 347. The 38 refers to n released or n detected of 58 released? Same issue in Fig 7 caption

We have added language to captions for figures 6 and 7 to clarify that ‘n’ refers to the number detected, not released.

38. Lines 354,405,501. Typo

L354 ‘released’ changed to ‘release’

L405 ‘term’ changed to “terms’

L501 ‘that’ changed to ‘than’

39. Line 367. Pond1 is a new term that has not been defined

‘Pond1 changed to ‘Pond 1’ and defined at first use (circa L211 in revised manuscript).

40. Line 368. ‘both BDAs’ – weren’t there 3?

Clarification language added that we were referring to BDAs 1 and 2 only.

41. Line 377. Poor topic sentence – reads like a table caption – that does not match the detection efficiency material that immediately follows.

The first (topic) sentence has been removed. It was unnecessary.

42. Line 379. The summary of detection efficiency was poor. Not really defined, but appears to just indicate whether fish were detected at individual antennas rather than some estimate of proportions detected of those in range of the antennas, which would be more traditional.

We removed the term ‘detection efficiency’ and replaced with ‘the percentage of fish detected’

43. Line 384. How was ‘detection rates’ defined?

We changed the language to clarify that we meant percentage of fish detected

44. Table 1. Terms need to be better defined, and an indication of when sites were combined or not. Also, check percentages 32/39 and 20/39 are 82% and 51%. . . . I did not check others.

Numbers have been checked and corrected as appropriate.

45. Line 403. Redundant with line 393?

L393 refers to jumps over either BDA 1 or BDA 2; L403 refers only to jumps over the taller BDA (BDA 2).

46. Line 416. Can data suggest or indicate preference? Better to just state coho jumped more frequently during the day. . . . Again, verb tense should probably not be present

Language corrected to remove present verb tense and the phrase “data suggest’ has been removed

47. Line 429. Why would these fish be expected to move upstream? Isn’t is likely/possible they originated from the Scott River? Also start the topic sentence with the result (These fish did not move upstream. . . .) and then contrast with the other results.

The first sentence was deleted so that the topic sentence starts with the result. We discuss why the fish probably wouldn’t be expected to move upstream and that they are likely from a different population in the discussion section.

48. Table 2. Any reason for switching from common to scientific names suddenly? The (100%) after every release group is not needed.

Names have been switched from scientific to common names. The 100% after every release group has been omitted.

49. Line 449. Still not clear where these fish were collected.

We clarified that they were collected in BDA Pond 1.

50. Discussion generally. Would benefit from some reorganization to directly address specific objectives and/or hypotheses as well as limitations, implication, etc. Could organize around the two species, perhaps, if the authors think the results or life histories are different enough to not lump together. This section overall feels like a rambling narrative that is not particularly well foreshadowed by the other manuscript sections. The authors also have a tic, where ‘suggest’ is repeated, I think almost a dozen times.

The ‘suggest’ tic has been acknowledged and removed. We don’t agree that it is a ‘rambling narrative’. The major results are very clear, the evidence is overwhelming that juvenile coho salmon and steelhead trout are able to cross these structures under that conditions we tested, and both the jumping route and the side channel passage were both well utilized. Extensive parsing of the data to find meaning or assist in interpretation was not necessary. The results speak for themselves. Because the results are so obviously clear, as both reviewers noted, the discussion focuses on the implications of those results in the context of management, that is, the practical application of these data.

51. Line 494. Not quite clear how this longish aside about culverts really applies. If the main point is to get to the jump height question, there is a fairly large literature that could be tapped. If I recall, the depth of the plunge pool is the key driver, since fish can accelerate from deeper sites better.

We left this discussion in place. We felt that this discussion will appeal to a wide range of readers who are practically-minded and interested in designing or permitting fish passage at natural restoration structures. It is a discussion of the potential practical application of the knowledge gained from this study.

52. Line 515. This is a pretty good statement for the front end of the Discussion.

No comment

53. Line 536. Not entirely clear that the Midwestern example was a beaver dam study.

We have clarified by inserting the phrase ‘across beaver dams’

54. Line 546. Age or direction of passage for the sucker study?

Sucker age and direction were not disclosed in the study

55. Line 549. The authors have not shown that the ponds ‘produced’ any fish! Yes, fish passed through that habitat, but the current study says nothing about rearing habitat. Even if the stream ran dry, couldn’t juveniles have exited before the drying?

We have changed the word ‘produced’ to ‘provided summer rearing habitat for’

56. Line 553. Similar issue here – the study does not ‘clearly’ demonstrate that breaching would have harmed the fish since downstream habitat was available. Need to be cautious about over interpretation here. The pond habitat was probably good and more is needed, but the experiment was not about these questions.

Changed ‘clearly’ to ‘arguably’ and edited the sentence to read ‘would arguably have been detrimental to the species due to the loss of summer rearing habitat that would have occurred when the pond drained.’

57. Line 563. What evidence is there in the experimental data that upstream juvenile passage is ‘less important’?

We deleted this sentence, as it was not germane to the discussion.

58. Line 565. Very long sentence with an uncertain message. Adult salmon can certainly jump 40 cm, and there are presumably many upstream pathways during higher discharge.

We shortened and edited the long sentence to read ‘The same can be said for adult salmon, especially coho salmon, which generally time their movements to coincide with high flows’

59. Line 570. Getting the sense that opposition to barrier removal was a motivating factor. That’s fine, just need to set this up at the front end.

We think it is pretty clear throughout the document that we are concerned about salmonid habitat degradation caused by beaver dam removal.

60. Line 586. Probably not intended, but the authors seem to be perpetuating the idea that connectivity is somehow independent from habitat complexity.

Changed ‘connectivity in fluvial systems’ to ‘removal of channel spanning structures that unreasonably restrict the movement of fishes’

61. Line 593. Scientific names missing

Scientific names have been added.

62. Line 596. Another very long sentence. It is not clear to me how this last word derives from the experimental results. How about a plug or two for more natural and analogue beaver dams?

63. Figure 3. Why not number the experiments chronologically?

They are numbered chronologically by year, and then for 2017, by level of importance.

64. Figure 5. Percents would be easier to interpret. Consider dropping ‘in each site’ from the y-axis caption.

We felt that the key points from the figures can easily be interpreted as the figure stands.

65. Figure 6. Add vertical lines for sunrise and sunset?

The times for sunrise and sunset are included in the figure caption

66. Figure 9. This has not caption and so not clear what all the notation means or what the point of the figure is.

The figure caption was inadvertently omitted. It has been included in the revised manuscript

67. Figures 10 and 11 could be combined. Using smoothed lines rather than bars would also make the diel cycles easier to see and interpret.

They could be combined, but we feel the data patterns are easier to interpret if each figure remains separate. We considered smoothed lines, but felt that the bars were easier to interpret.
